# Roles and Mechanisms of the Protein Quality Control System in Alzheimer’s Disease

**DOI:** 10.3390/ijms23010345

**Published:** 2021-12-29

**Authors:** Yaping Liu, Runrong Ding, Ze Xu, Yuan Xue, Dongdong Zhang, Yujing Zhang, Wenjie Li, Xing Li

**Affiliations:** Department of Nutrition and Food Hygiene, College of Public Health, Zhengzhou University, Zhengzhou 450001, China; liuyaping_tang@163.com (Y.L.); dingrr123@163.com (R.D.); ze1995@163.com (Z.X.); xueyuansnow@163.com (Y.X.); dongdongzhang23@163.com (D.Z.); 15093458874@163.com (Y.Z.); lwj@zzu.edu.cn (W.L.)

**Keywords:** Alzheimer’s disease, protein quality control, endoplasmic reticulum stress, autophagy–lysosome, ubiquitin–proteasome

## Abstract

Alzheimer’s disease (AD) is characterized by the deposition of senile plaques (SPs) and the formation of neurofibrillary tangles (NTFs), as well as neuronal dysfunctions in the brain, but in fact, patients have shown a sustained disease progression for at least 10 to 15 years before these pathologic biomarkers can be detected. Consequently, as the most common chronic neurological disease in the elderly, the challenge of AD treatment is that it is short of effective biomarkers for early diagnosis. The protein quality control system is a collection of cellular pathways that can recognize damaged proteins and thereby modulate their turnover. Abundant evidence indicates that the accumulation of abnormal proteins in AD is closely related to the dysfunction of the protein quality control system. In particular, it is the synthesis, degradation, and removal of essential biological components that have already changed in the early stage of AD, which further encourages us to pay more attention to the protein quality control system. The review mainly focuses on the endoplasmic reticulum system (ERS), autophagy–lysosome system (ALS) and the ubiquitin–proteasome system (UPS), and deeply discusses the relationship between the protein quality control system and the abnormal proteins of AD, which can not only help us to understand how and why the complex regulatory system becomes malfunctional during AD progression, but also provide more novel therapeutic strategies to prevent the development of AD.

## 1. Introduction

Alzheimer’s disease (AD) is an aging-related neurodegenerative disorder accompanied by memory loss, cognitive impairment, synaptic damage and behavioral changes [1]. As one of the most principal forms of dementia, it accounts for 70% of patients with dementia [2,3,4,5]. Currently, it is estimated that approximately 47 million people suffer from dementia worldwide [6] and with the aggravation of global population aging, the number of people with dementia cases will escalate to 74.7 million by 2030 and 131.5 million by 2050 [7,8]. Presently, growing evidence indicates that the main clinical manifestations of AD mostly occur after 65 years of age [9], including aphasia, apraxia, agnosia, an incapacity for discernment, and changes in personality and behavior and culminating in an individual’s death [10,11,12].

However, it should be noted that as the most common chronic neurological disease in the elderly, the challenge of AD treatment at present is the absence of an effective combination of sensitive biomarkers for early diagnosis. Some studies have shown that the pathological biomarkers of AD mainly include extracellular deposition of senile plaques (SPs) composed of amyloid-β protein (Aβ) and intracellular accumulation of neurofibrillary tangles (NFTs) consisting of hyperphosphorylated Tau (p-Tau), as well as neuronal loss in different brain regions. In fact, patients have shown a sustained disease progression for at least 10 to 15 years before these biomarkers are detected [13]. Thus, the challenge of early diagnosis of AD is far from being solved. Furthermore, the current approaches to treating AD are still severely lagging. In spite of drugs aimed at relieving the symptoms of AD, patients have been widely studied for a long time, which has largely been ineffective or inconclusive ultimately due to a variety of reasons [14]. For example, the drug that targets Aβ has been proved to provide symptomatic relief for only the initial 1–2 years, but is incapable of preventing or delaying the progression of the AD pathology fundamentally [8,15]. With the changes in dietary structure and the increase of life expectancy, AD has become the most devastating neurodegenerative disorder characterized by a high morbidity rate and mortality [14,16]. Simultaneously, it also brings a heavy economic burden [2], and even has the potential to evolve into a global public health concern if left unchecked [10,17,18,19,20].

Currently, mounting evidence indicates that the accumulation of abnormal proteins such as Aβ and p-Tau is closely associated with the dysfunction of the protein quality control system in the brain [21]. Particularly in the early stage of AD, the synthesis, degradation and removal of essential biology components in the protein quality control system changes, suggesting that the protein quality control system may be a new and potential target of AD therapy [22]. Hence, it is of great significance to further explore the relationship between the protein quality control system and the pathogenic proteins of AD. This review mainly focuses on the endoplasmic reticulum system (ERS), autophagy–lysosome system (ALS) and ubiquitin–proteasome system (UPS), and discusses the interrelation between the protein quality control system and the abnormal proteins of AD, which can not only help us understand how and why this complex regulatory system becomes malfunctional during AD progression, but also provide more novel therapeutic strategies to prevent the development of AD.

## 2. The Protein Quality Control System

**Figure 1:**
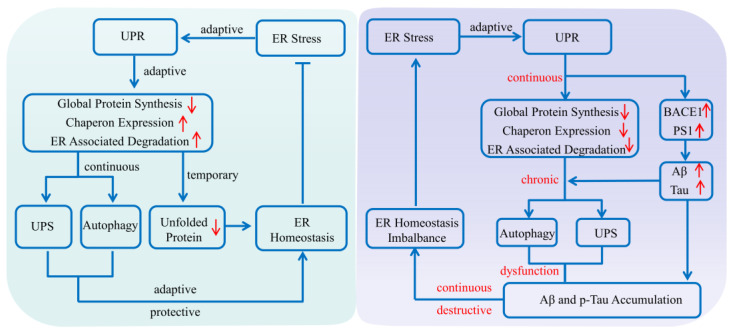
Protein quality control system affecting Alzheimer’s disease. The endoplasmic reticulum system, autophagy–lysosome system and ubiquitin–proteasome system are the three main regulatory pathways in maintaining normal cell metabolism and avoiding protein dysfunction. Once abnormal protein accumulates in the brain, the unfolded protein response is initially provoked to produce normal proteins by upregulating the expression of molecular chaperones, while reducing the accumulation of misfolded proteins via inhibiting the synthesis of total proteins. If the endoplasmic reticulum system fails to refold the abnormal protein for some reason, the molecular chaperones will deliver the abnormal proteins to the autophagy–lysosome system or the ubiquitin–proteasome system, where the abnormal proteins can be effectively degraded. While during AD progression, all the abnormal expression and impaired function of key components of these pathways, as well as defects in the proteins’ interplay, could induce dysregulation of proteostasis and contribute to AD pathogenesis. (Aβ: amyloid-β protein; BACE1: β-amyloid precursor protein cleaving enzyme 1; ER: endoplasmic reticulum; PS1: presenilin 1; p-Tau: hyperphosphorylated Tau; UPR: unfolded protein response; UPS: ubiquitin–proteasome system). The upward red arrow indicates up-regulation of expression, while the downward red arrow indicates downregulation of expression. The blue arrow indicates the activation of process, while the blue T arrow indicates the inhibition of process.

As a collection of pathways that regulates proteins’ life cycles including their synthesis, folding, assembly, degradation and reversal, the protein quality control system mainly consists of the ERS, ALS and UPS [23]. It plays an important role in maintaining normal cell metabolism and avoiding protein dysfunction, especially in the physiological and pathological processes of AD [22]. Various evidence shows that when abnormal protein accumulates, as an adaptive response of the ERS, the unfolded protein response (UPR) is provoked to produce normal proteins by up-regulating the expression of molecular chaperones, while reducing the accumulation of misfolded proteins via decelerating the synthesis of total proteins [24]. In addition, the adaptive and protective interactions between the ERS and UPS can help cells to clear toxic protein aggregation, reduce the imbalance in the endoplasmic reticulum lumen, and restore cell homeostasis [1]. However, the continuous imbalance of endoplasmic reticulum homeostasis, as well as autophagy dysfunctions could mediate the gradual transformation from the ERS to autophagy, that is, from an adaptive and protective state to a persistent and destructive condition [24]. For example, if the ERS fails to refold the abnormal proteins for some reason, the molecular chaperones would deliver the abnormal proteins to the autophagy–lysosome system or ubiquitin–proteasome system, where the abnormal proteins can be effectively degraded [22]. During the progression of AD, due to the abnormal expression and impaired function of key components of these pathways, as well as deficits in protein homeostasis, the abnormal protein would be encapsulated in the proteasome and lysosome to form the endosome and be degraded unsuccessfully, which finally induces dysregulation of proteostasis and pathological damage (Figure 1).

Increasing evidence shows that the deposition of abnormal proteins including Aβ and p-Tau in AD is associated with dysfunction of the protein quality control system in the brain [22]. In particular, during AD progression, abnormal expression and impaired function of key components, as well as defects in the proteins’ interplay could induce dysregulation of proteostasis and contribute to AD pathogenesis [22]. Therefore, it is of great importance to explore the interrelationship between the protein quality control system and the pathogenesis of AD, which will help us to further understand the mechanisms and consequences of proteostasis dysregulation in detail, and even provide a potential novel therapeutic strategy for AD.

### 2.1. Endoplasmic Reticulum System and AD

Endoplasmic reticulum is a principal eukaryotic organelle responsible for protein folding, modification and secretion, in addition to lipid synthesis and calcium storage [25,26]. Endoplasmic reticulum dysfunction caused by genetic mutations or environmental stimuli can lead to the accumulation of unfolded and misfolded proteins [27], which will trigger the UPR, subsequently resulting in a series of downstream reactions [28,29]. Current studies have shown that the UPR can not only reduce total protein synthesis by altering the intracellular transcription and translation processes [11], but also enhance the protein folding function and prevent the output of unfolded or misfolded proteins by up-regulating the molecular chaperones in the endoplasmic reticulum [27]. Moreover, it can also facilitate abnormal protein degradation via the ER-associated protein degradation (ERAD) pathway [11].

Under normal physiological conditions, there are three transmembrane ER-proximal sensors including protein kinase RNA-like ER kinase (PERK), activating transcription factor 6 (ATF6), and inositol-requiring protein 1 (IRE1) [30]. These ER sensors can form an inactive complex with the 78 kDa glucose-regulated protein (GRP78) [31], which acts as an ER chaperone that participates in the polypeptide translocation, thus being sequestered [30]. Nevertheless, under ERS conditions, the accumulating unfolded or misfolded proteins preferentially combine with GRP78 and activate the signaling pathways including the phosphorylation of PERK and IRE-1, as well as the translocation of ATF6 to the Golgi [26,31], which can regulate the expression of chaperones, decrease the accumulation of abnormal proteins, restore endoplasmic reticulum homeostasis and maintain cell functions [11,28]. Therefore, it can be concluded that in the early stage of the ERS, the UPR are mainly occurring to reduce the abnormal proteins in the ER by inhibiting the overall synthesis of proteins and clearing abnormal protein aggregations [29,32], so as to maintain the homeostasis of the ER [33]; However, if the ERS is persistent and unresolvable, the UPR will hyperactivate and even induce cell dysfunction and apoptosis [11,26,34].

**Figure 2:**
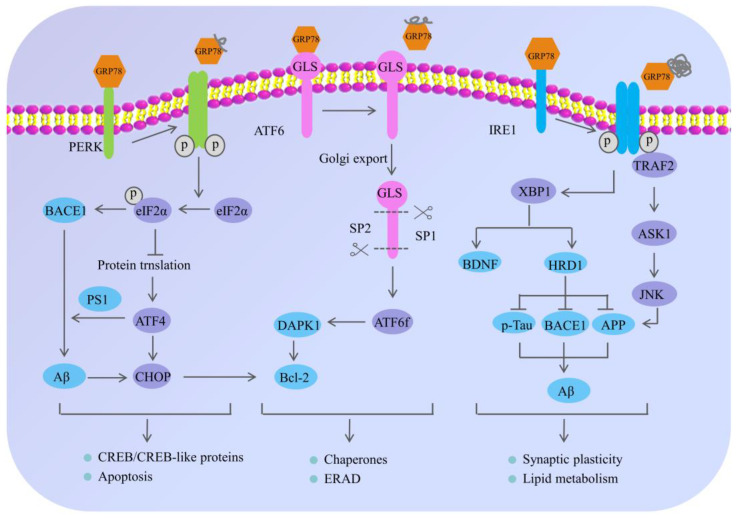
The mechanism of endoplasmic reticulum stress and its potential role in Alzheimer’s disease. Under normal physiological conditions, the ER sensors including PERK, ATF6 and IRE1 are inactivated through the interaction with 78 kDa glucose-regulated protein (GRP78); however, the misfolded proteins preferentially bind to GRP78, causing the dissociation of GRP78 from PERK, IRE1 and ATF6, eventually resulting in the phosphorylation of PERK and IRE-1, and the translocation of ATF6 to the Golgi. The activation of these signaling pathways regulates the expression of chaperones and decreases the accumulation of abnormal proteins, which can restore endoplasmic reticulum homeostasis. In neurons, under chronic ERS, the sustained activation of PERK leads to eIF2α phosphorylation, which not only influences the neuronal plasticity through protein synthesis inhibition, but also upregulates the expression of BACE1 and ATF4. Meanwhile, the BACE1 can be involved in the production of Aβ, and the ATF4 can further trigger cell death by upregulating the CHOP. Moreover, the adaptive activation of IRE1α leads to XBP1 splicing, which directly or indirectly participates in AD pathogenesis. On one hand, XBP1 can increase the degradation rate of key AD proteins—APP, BACE1 and p-Tau through inducing the E3 ubiquitin–ligase HRD1. On the other hand, the specific XBP1s’ splicing by IRE1 can also increase the generation of neurotrophic factor BDNF; however, the continuous activation of IRE1α leads to the preferential phosphorylation of TRAF2 and the inhibition of XBP1s splicing, which can further activate the downstream JNK signaling pathway and cause neuronal apoptosis. In addition, ATF6 is localized at the ER in physiological conditions and encodes a bZIP transcriptional factor in its cytosolic domain. While undergoing sustained ERS, ATF6 can translocate to the Golgi apparatus where it is processed by site 1 and 2 proteases releasing its cytosolic domain (ATF6f), and further controlling the upregulation of UPR target genes. The arrow indicates the activation of process, while the T arrow indicates the inhibition of process.

Although AD has attracted much attention, its specific pathogenesis has not been fully elucidated [25,35,36,37,38]. Recently, a growing body of evidence demonstrates that the deposition of SPs and the formation of NFTs are not only salient features of AD, but linked to pathological ERS [39,40,41], highlighting the interrelationship of the ERS and AD [25,38,42,43]. In particular, the ERS is closely related to the production and accumulation of Aβ [17,44]. Under normal circumstances, the sequential cleavage of APP by α-secretase and γ-secretase occurs without the generation of Aβ. While in the pathological state of AD, the APP can be sequentially hydrolyzed by β-secretase and γ-secretase, and can then generate Aβ and induce toxicity cascade effects [45]. Liu et al. found that under the adaptive and protective ERS condition, the level of APP decreased in AD model cells induced by tunicamycin. In addition, the autopsy results of AD patients showed that the level of ER stress in the brain tissue had increased [46], indicating that the ERS might play an important role in AD [4,47,48] (Figure 2).

PERK, a type I transmembrane protein located in the ER, exerts serine/threonine kinase activity through its cytoplasmic domain [27]. During the early stage of the ERS, adaptive activation of PERK is a protective cellular mechanism [45]; however, persistent activation of PERK causes hyperphosphorylation of eIF2α at Ser51 [49], which can inhibit general translation initiation, lead to a reduction of critical memory proteins [17,50], further resulting in cognitive disorder and neurodegeneration [26,45,51]. Moreover, long-term sustained phosphorylation of eIF2α can also specifically up-regulate the expression of the β-amyloid precursor protein cleaving enzyme 1 (BACE1), which is a key enzyme responsible for initiating the generation of Aβ and promoting the formation of Aβ. Additionally, the hyperphosphorylated eIF2α can also activate the activating transcription factor 4 (ATF4). On one hand, ATF4, as a repressor of the cAMP response element binding protein (CREB)-dependent transcription, is responsible for long-term memory and synaptic plasticity [17,45], while the overexpression of ATF4 would severely impair memory function in AD. On the other hand, ATF4 mediates the abnormal processing of APP and promotes the excessive deposition of Aβ by upregulating the expression of presenilin 1 (PS1), which is an important cofactor for the production of Aβ. In addition, ATF4 can also act as a promoter of glycogen synthase kinase-3β (GSK-3β) expression to promote Tau hyperphosphorylation in AD patients [52]. Obviously, it suggested that the continuously activated PERK/eIF2α pathway may contribute to AD pathogenesis and cognitive impairments in many ways [45,53,54].

IRE1 is a transmembrane sensor kinase and an endoribonuclease that mediates both adaptive and proapoptotic pathways under ERS conditions [25]. The adaptive activation of IRE1α can lead to the splicing modification of XBP1 (a transcription factor of the leucine zipper family), which can up-regulate the expression of genes related to protein folding and promote the correct folding of proteins [32]. Moreover, XBP1 can increase not only the degradation rate of key AD proteins (APP, BACE1 and p-Tau) by inducing the E3 ubiquitin–ligase HRD1, but also the generation of neurotrophic factor BDNF [55]. Despite the initial activation of IRE1 signaling that may decrease the accumulation of abnormal proteins in AD, the continuous activation of IRE1 would mediate the phosphorylation of tumor necrosis factor receptor-associated factor 2 (TRAF2) and the inhibition of XBP1 splicing, which can trigger the c-Jun NH2-terminal kinase (JNK) signaling pathway and cause neuronal apoptosis [55,56]. To be specific, activated IRE1α on the ER membrane interacts with TRAF2, thus activating the following reactions: (1) it may recruit apoptosis signal-regulating kinase 1(ASK1), also known as MAP kinase, leading to activation of the mitochondria-dependent caspase apoptosis pathway [57,58]; (2) activated ASK1 further phosphorylates JNK and Bcl-2, eventually inducing apoptosis of nerve cells and aggravating nerve injury [59]; and (3) activated JNK can also in turn phosphorylate TRAF2, causing procaspase-12, which was originally bound to TRAF2, to be dissociated from the complex and cleaved after oligomerization to form active Caspase-12, leading to the occurrence of cell apoptosis [59]. Previous research showed that genetic ablation of the RNase domain of IRE1 in the nervous system significantly reduced the content of amyloid β oligomers, improved cognitive function and attenuated astrocyte activation [25]. At the molecular level, the deletion of IRE1 reduced the expression of APP in the cortical and hippocampal areas of AD mice [59].

Unlike PERK and IRE1 that belong to the endoplasmic reticulum type I transmembrane proteins family, ATF6 belongs to the endoplasmic reticulum type II transmembrane proteins family [56]. Once ERS occurs, GRP78 dissociates from ATF6 and is transported to the Golgi, where the active form of the ATF6 fragment with transcriptional activity is formed after the hydrolysis of protease site-1 and site-2. Then the ATF6 fragment is further transferred from the cytoplasm to the nucleus and regulating the expression of various genes related to ERS, such as GRP78 and protein disulfide isomerase, which promote protein folding and relieve ER pressure [24]. Meanwhile, the ATF6 cooperates with IRE1α to facilitate Xbp1-mediated transcription [60], and the ATF6 and XBP1 both activate PERK/eIF2α signaling, which suggests that there is an interaction among the three pathways of the URP. In addition, Du et al. found that ATF6 can reduce the expression of BACE1 by regulating the activity of the BACE1 promoter, thereby reducing the production of Aβ1-42, improving the learning and memory ability of mice and slowing down the pathological process of AD [55]. Some other studies have shown that the hyperphosphorylation of ATF6 can activate the death-associated protein kinase 1 signaling pathway, promote the phosphorylation of Bcl-2, activate autophagy, and accelerate apoptosis [48,56].

Abnormal ERS mechanisms are not only associated with AD, but also with other neurodegenerative diseases, such as prion diseases, Parkinson’s disease, and amyotrophic lateral sclerosis [48]. Consequently, the targeting of ERS in AD may be an interesting therapeutic approach, which can help us to further understand the pathogenesis of AD and provide us with a novel direction to prevent and treat AD in terms of the regulation mechanisms of ERS.

### 2.2. Autophagy–Lysosomal System and AD

Autophagy, also known as “self-eating”, is a highly conserved lysosomal degradation pathway that is responsible for the delivery and digestion of cellular contents, organelles and misfolded proteins in the cellular catabolic processes [61,62,63]. Based on the different degradation mechanisms [64], autophagy is classified into three general types in most mammalian cells: macroautophagy, microautophagy and chaperone-mediated autophagy (CMA) [3,61,65]. Moreover, it should be noted that the three forms of autophagy are not exactly the same, though they share similar functions [62], which are summarized as follows: (1) macroautophagy in which the cytoplasmic component is engulfed by autophagy vacuoles and degraded by proteases after fusion with lysosomes; (2) microautophagy in which the cytoplasmic components are directly engulfed by lysosome through invagination or protrusion; and (3) CMA in which the cytoplasmic proteins are selectively delivered into lysosome by recognizing their specific motifs through lysosomal receptors [66]. Among these, macroautophagy, simply referred to as autophagy, represents the vast majority of autophagic processes [61,67]. Different from microautophagy and CMA that mainly degrade small molecules [68], macroautophagy refers to a degradation pathway that digests large protein aggregates or damaged organelles [64], and is vital to organ development and cellular function [68].

Macroautophagy begins by encasing the bulk cytoplasm or selected organelles with a double membrane of multiple proteins [66], which then becomes a double-membrane vesicle that engulfs the protein aggregates and damages the organelles through the extension of an isolation membrane, also known as the phagophore [61]. This phagophore continues to expand and engulf intracellular cargos, while sequestering inclusions in a double membranous autophagosome [64]. The autophagosomes are formed randomly in the cytoplasm and then transported along microtubules in a dynein-dependent manner towards the microtubule-organizing center [64]. Once arrived at the center, the autophagosomes may either fuse with endosomes to generate amphisomes, which may eventually merge with lysosomes to dispose of their cargo; or they may fuse directly with lysosomes to form autolysosomes [61], and then be degraded by the specific proteolytic enzymes in the lysosomes. Subsequently, the lysosomal permeases and transporters export amino acids and other by-products of degradation back to the cytoplasm for the synthesis of macromolecules, thus participating in metabolisms [64,66].

**Figure 3:**
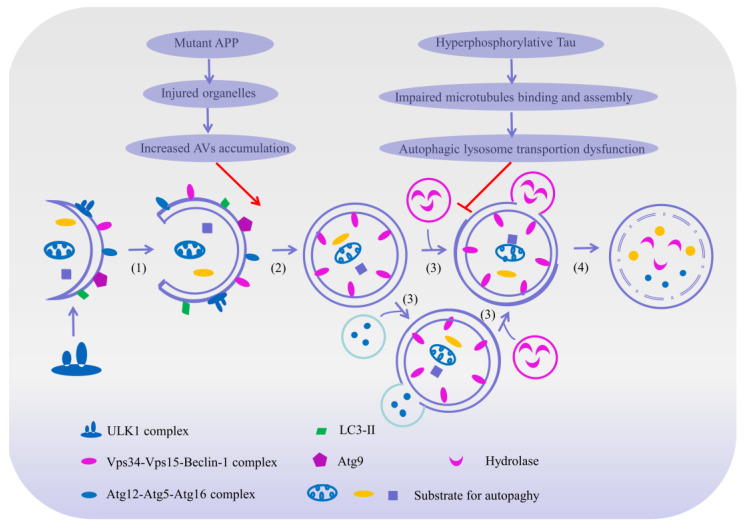
The mechanism of autophagy and its potential role in Alzheimer’s disease. Macroautophagy can be broken down into the following essential steps: (1) Initiation: macroautophagy begins by encasing the abnormal protein or selected organelles with an intracellular bilayer membrane structure to form a primary cup-shaped compartment containing a bilayer membrane called a phagophore. (2) Extension and completion: with the help of an Atgl2-Atg5-Atg16 complex, Atg8/LC3 and Atg9, the phagophore further engulfs the protein aggregates and impaired organelles through the extension and isolation of membranes, and finally generates a spherical double-membraned structure called an autophagosome. (3) Fusion: some autophagosomes fuse with an endosome to form an amphisome to dispose of its cargo, and others merge directly with lysosome to form an autolysosome. (4) Maturation and degradation: the amphisome and autolysosome are digested by various lysosomal hydrolases into amino acids and other small molecules, and subsequently transported back out to the cytoplasm for the synthesis of macromolecules thus taking part in metabolism. Nevertheless, the mutations of the APP gene can cause organelles’ damage, leading to an increased production of autophagy vesicles. In addition, the hyperphosphorylation of the Tau protein can impair the binding and assembly of microtubules, thereby impeding the formation and transportation of autophagosomes. When the maturation and degradation of autophagosomes are inhibited, the autophagic pathways will be damaged and a consistent accumulation of intracellular Aβ and Tau will take place, therefore possibly leading to AD. The arrow indicates the activation of process, while the T arrow indicates the inhibition of process.

Autophagy, as a complementary mechanism for the proteasome system, is responsible for the elimination of misfolded proteins, damaged organelles and long-lived macromolecules by an essential lysosomal pathway in the cellular catabolic process [62,63,64,66], which exerts an essential cytoprotective mechanism in maintaining cellular homeostasis, energy balance and cellular defense [67,69] Although autophagy is present in all cell types, it is more important to neurons [68], as these cells are more sensitive and active to the stresses caused by damaged organelles or misfolded proteins than somatic cells, and are not easily regenerated once being eliminated [67]. Therefore, autophagy is now recognized as one of the contributors to neuronal survival and death in neurodegenerative diseases, and particularly, mounting evidence has implicated that autophagy dysregulation may play a critical role in the pathogenesis of AD [64,65]. Generally speaking, in a normal physiological state, autophagy vesicles cooperate with lysosomes to degrade abnormal proteins in healthy neurons [70]; however, in the early stage of AD, the mass production of abnormal proteins has been shown to cause damage to the autophagy–lysosome pathway [66] and with the progression of AD, autophagic dysfunction occurs continuously and autophagic vesicles accumulate steadily, which further disturbs the turnover of other molecules and aggravates the neuronal dysfunctions in AD [66]. Furthermore, autophagy dysfunctions lead to the over-accumulation of Aβ and p-Tau protein in neurons, which might directly disturb neuronal homeostasis and accelerate cell apoptosis [66]. Meanwhile, it might also affect the expression and function of other important molecules such as BACE1, apolipoprotein E (ApoE) and impair mitochondria function, which may further accelerate the progress of AD [66] (Figure 3). More interestingly, some studies have shown that ahead of the formation of the SPs and NFTs, the expressions of lysosome-related components are significantly increased, suggesting that the lysosome system is activated before the pathological alteration [7,66,71].

The substantial evidence available manifests the idea that autophagy is involved in the processing of Aβs. It is well known that intraneuronal Aβ is generated predominantly via sequential cleavages of APP by β-secretase and γ-secretase complexes [66]. The APP belongs to the type I transmembrane protein family, which is widely distributed in various tissues, especially in the axons and dendrites of neurons [70]. The β-secretase cleaves the APP into soluble APPα and β-carboxyl-terminal fragment (β-CTF), and the γ-secretase continues to dissolve the β-CTF into various types of Aβ [72]. Yu et al. found that autophagic vacuoles (AVs) in mice hepatocytes with an overexpression of APP contained a large number of APP, β-CTF and BACE1, suggesting that AVs may be one of the potential sites for the processing of Aβs [73]. Subsequent studies have further elucidated that AVs in the brains of AD patients also contain large amounts of APP, β-CTF and γ-secretase complexes, demonstrating that autophagy was activated during the course of AD, thus leading to an amplification of AVs and production of large amounts of Aβ [73]. Consequently, all the above studies revealed that autophagy may participate in the turnover of Aβ. Thus, we have assumed that not only may the AVs degrade the encapsulated APP into Aβ, but the β-CTF in the endosome might also be delivered to the autophagosome and hydrolyzed by γ-secretase to produce more Aβ.

In addition, autophagy also takes part in the clearance of Aβ. In a physiological state, AVs that are rich in Aβs are transported retrogradely to the neuronal soma where they can fuse with the lysosome and become degraded efficiently by acidified proteases [74,75,76]; however, with the progression of AD, the hyperphosphorylation of Tau impairs microtubule binding and assembly, further impedes the AV-lysosome fusion and retrograde transportation, which in turn leads to a more rapid accumulation of AVs in the dystrophic neurites [66]. More recent studies have found that a large number of autophagosomes and other types of AVs containing APP were accumulated in the cerebral cortex and hippocampal swelling axons of AD patients, as well as model mice [77,78], indicating an impaired clearance function of AVs in AD brains. Retained AVs cannot be degraded by lysosomes effectively, which may result in Aβ accumulation in cells and accelerate the pathological processes of AD. Additionally, impairment in lysosomal membrane integrity also disrupts autophagy–lysosome function, which may further interfere with the intracellular Aβ degradation and greatly exacerbate neuron dysfunction [66,74].

As another pathological biomarker of AD, p-Tau has aroused people’s widespread concern and heated discussion [79,80]. Especially recently, although the amyloid cascade hypothesis has been widely accepted in AD research for many years, clinical Aβ-targeting strategies have consistently failed to improve or prevent AD, therefore the research focus of AD has recently shifted to the role of Tau [7], and a growing body of evidence suggests that Tau indeed has unique roles that are independent of Aβ in AD. Tau is predominantly expressed in neurons, and also can be found in the extracellular environment [7,81]. In mature neurons, Tau is concentrated in the axonal where it interacts with microtubules to stabilize the microtubules and promote microtubule assembly [82]. In addition, Tau is also involved in axon elongation, maturation and axonal transport via different mechanisms [7,83]. Some studies have indicated that hyperphosphorylated Tau appears in the early stages of AD, and that it is more prone to aggregation and tangles [14,84], which might in turn impair axonal transport, mitochondrial function, and cytoskeletal dynamics in a manner that is independent from Aβ [7]. Interestingly, most of the processes in the autophagy, especially the autophagosome transport, primarily depend on the normal function of microtubules, and Tau protein is associated with microtubule binding and assembly [66]. Moreover, NFTs composed of p-Tau protein have been identified in certain lysosome storage disorders (LSDs) and AD, which potentially represent one of the probable pathogenic mechanisms of AD [65]. All these emerging studies strongly suggest that the post translational modification of Tau is tightly linked to autophagy–lysosomal reactions in AD.

Furthermore, under normal conditions, Tau is preferentially degraded by the macroautophagy pathway [14,85]; however, a majority of Tau is found in the form of hyperphosphorylation in the axonal of AD models [66], which shows it is not only dysfunctional, but also pathogenic as it can induce microtubule malformations, disrupt the microtubule-mediated transport, further impair the fusion of autophagosome and lysosome [66], while leading to the accumulation of AVs [78]. In turn, the accumulation of AVs also accelerates the aggregation of p-Tau to a large extent [85,86], therefore forming a vicious circle and causing lysosomal dysfunction and neuronal death in the case of AD [87,88]. In addition, it has been demonstrated that soluble and aggregated forms of Tau can be degraded via autophagy, whereas the inhibition of autophagy can promote Tau aggregation and toxicity. All the above mentioned reports indicated that Tau is deeply involved in the autophagic pathway through its function in microtubule assembly [66]. Moreover, consistent with these viewpoints, deletion of genes that are essential for autophagy resulted in the accumulation of protein aggregates and neuronal cell death, which further proved that constitutive autophagy is essential for both normal protein turnover and neuron survival [65].

Consequently, there exists a tight link between neural autophagy and AD, and autophagy dysfunction plays an important role in the pathological process of AD. Intriguingly, inhibiting the over-accumulation of Aβ and p-Tau via regulating autophagy could be a potential therapeutic strategy for AD.

### 2.3. Ubiquitin–Proteasome System and AD

**Figure 4:**
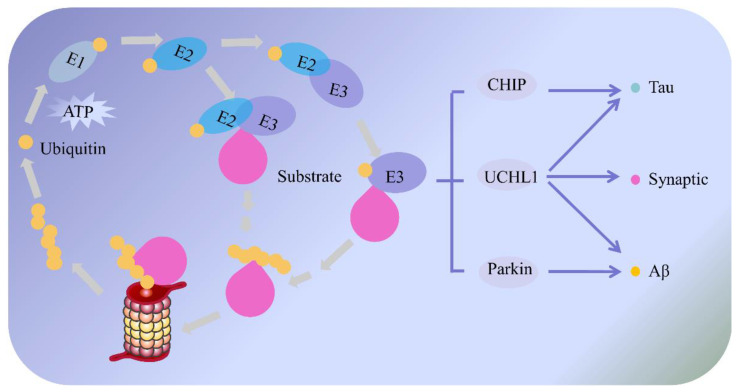
The mechanism of the ubiquitin–proteasome system and its potential role in Alzheimer’s disease. The ubiquitin–proteasome system includes two successive steps: ubiquitination and proteasome degradation. Initially, ubiquitin is activated by a ubiquitin-activated enzyme (E1) in an ATP-dependent process. Then, the activated ubiquitin is transferred to a ubiquitin-conjugating-enzyme (E2). Finally, E2 transfers the ubiquitin moiety from E1 to the target protein, which is recognized and tagged by the ubiquitin–protein ligase (E3). Following this tagging, the polyubiquitinated substrates are transported to the 26S proteasome for degradation by the proteasome. Meanwhile, the UPS serves as a critical way to remove the accumulation of abnormal proteins and prevent the progression of AD. For example, as an important E3 ligase in the UPS, Parkin not only interacts directly with Aβ and decreases its accumulation, but also indirectly increases the clearance of Aβ via the proteasomal dependent pathway. CHIP is an E3 ubiquitin ligase that ubiquitinates Tau protein, thereby promoting the degradation of abnormally phosphorylated Tau protein. Moreover, the UCHL-1 is an E3 ligase and deubiquitination enzyme with the function of degrading abnormal protein and improving synaptic plasticity.

Different from the way that the lysosome autophagy system clears long-lived proteins and intracellular organelles, the UPS represents the main non-lysosomal mechanism for short-lived protein degradation [89,90], and it also helps to maintain overall proteostasis by preventing the accumulation of abnormal proteins in eukaryotic cells [91,92]. In addition to protein quality control, the UPS has also been deeply involved in many crucial cellular biological processes via the degradation of a huge number of regulatory proteins [93,94]. For example, it has been shown that the UPS is a key regulator of almost all metabolic pathways, including proliferation, differentiation, apoptosis, cell cycle, DNA repair, epigenetic control, signal transduction, transcriptional regulation, inflammation, synaptic plasticity and antigen processing [89,93,94,95,96,97,98].Generally, the UPS mediates the removal of damaged soluble proteins and degradation of short-lived regulatory proteins by two successive steps [89]: (1) ubiquitination, which refers to an enzymatic post-translational modification of damaged or misfolded proteins [91] and is tagged by covalent attachment of multiple ubiquitin molecules [93]; and (2) the proteasome degradation [91,99], that is, the tagged protein is then transferred to the proteasome complex for degradation and eventually releases the reusable ubiquitin [93]. Specifically speaking, ubiquitination is a well-known three-step cascades reaction [93], including activation, conjugation, and ligation [91]. Initially, ubiquitin, a highly evolutionarily conserved 76-residue polypeptide [93], is activated by an ubiquitin-activated enzyme (E1) in an ATP-dependent manner [91,100]. Then, the activated ubiquitin is transferred to the ubiquitin-conjugating-enzyme (E2). Subsequently, E2 transfers the ubiquitin moiety from E1 to target proteins, which is recognized and tagged by the ubiquitin–protein ligase (E3) [91]. In this process, E3 enzymes play key roles in the ubiquitin-mediated proteolytic cascade through a recognition of and reaction with specific substrates [93]. Finally, the poly-ubiquitinated substrates are transported to the 26S proteasome for further degradation and are subsequently broken down into short peptides and amino acids that are later recycled for new protein synthesis [101,102]. The ubiquitin molecules are then recycled into the next proteasome pathway [89,91,100] (Figure 4).

Recent studies have shown that as an essential cellular protective mechanism, the UPS has attracted much attention in the pathogenesis of AD [101], as it can regulate the generation and accumulation of Aβ via multiple pathways and mechanisms [103,104]. On one hand, there is accumulating evidence indicating that Aβ could be one of the substrates of the proteasome complex, and UPS dysfunction has a significant effect on Aβ aggregation [89,105]. Particularly, the UPS dysfunction is linked with the excessive accumulation of ubiquitination-associated proteins in the brain of AD patients, which may further affect the generation and degradation of Aβ, and finally lead to the abnormal deposition of Aβ [101]. For instance, some studies have indicated that ubiquilin-1 is a ubiquitin protein that inhibits neuronal APP aggregation in vitro and vivo [72,106,107,108]; however, in the brains of AD patients, ubiquilin-1 levels were significantly reduced, along with the accumulation of APP, which in turn promoted the production and deposition of Aβ. Another study found that the treatment of proteasome inhibitor in primary cultured cortical neurons and astrocytes remarkably inhibited the activity of 26S proteasome, and significantly reduced the degradation of Aβ42, indicating that 26S proteasome was directly involved in the degradation of Aβ42. On the other hand, as the competitive substrates of the proteasome [101], the over-accumulation of Aβ could also inhibit the proteolytic activity of the 26S proteasome to some extent [89]. Especially in the case of AD patients, the continuous increase of Aβ is able to influence the expressions of ubiquitin-protein conjugates and ubiquitin-activated enzyme E1 in neurons, as well as compete against natural proteasomal substrates, thereby leading to the proteasomal dysfunctions [89,101,109]. To sum up, the UPS dysfunction can lead to abnormal aggregation of Aβ by inhibiting Aβ degradation and promoting the hydrolysis of the amyloid precursor protein. Meanwhile, Aβ has also been shown to inhibit the activity of UPS proteasome. Thus, a vicious circle is formed between UPS dysfunction and the aggregation of Aβ.

In addition to the extracellular deposition of SPs composed of Aβ, another pathological hallmark of AD is the intracellular accumulation of NFTs consisting of p-Tau [110]. A growing body of evidence suggests that dysfunctions of the UPS, as well as the over-expression p-Tau, are intensively correlated [111,112]. Studies have demonstrated that the accumulation of p-Tau at pre- and post-synaptic terminals was directly associated with and increased expression of ubiquitinated substrates and proteasome elements in the brains of AD patients [89,111], suggesting that p-Tau may be a potential biomarker of UPS impairment and synaptic dysfunction [101]. Moreover, in vitro experiments using Tau aggregates isolated from human AD brains confirmed that the activity of proteasome could be reduced by aggregated Tau, whereas non-aggregated Tau had no such effect [89], which further verified the interaction between aggregated Tau and proteasome. Additional data illustrated that inhibition of the proteasome resulted in a reduced Tau degradation, while the incubation of proteasome enhanced the degradation of Tau, which directly demonstrated the involvement of the UPS in Tau turnover in vitro [89]. Furthermore, the ubiquitin C-terminal hydrolase (UCHL1), as a UPS regulator, is mainly expressed in neurons, and acts not only as an E3 ligase, but also a deubiquitination enzyme that stabilizes monoubiquitin proteins [89,101]. A great number of studies have shown that the reduction of cytosolic UCHL1 is linked to AD progression [113], and to be precise, UCHL1 owns a function of the degrading abnormal Tau protein. In particular, soluble UCHL1 and the number of NFTs in the brain of AD patients are inversely proportional [114]. Besides, the carboxyl terminus of the heat shock protein 70 (Hsp70) interacting protein (CHIP) is another E3 ubiquitin ligase with the ability to ubiquitinate Tau protein, thereby promoting the degradation of abnormally phosphorylated Tau. Improving the level of CHIP can reduce the aggregation of the Tau, as well as the formation of NFTs, which make it a possible candidate for the treatment of AD [115]. Additionally, Keck et al. found that paired helical filaments assembled by the Tau proteins in AD brains were co-precipitated with the proteasome, which may in turn cause proteasomal dysfunctions [116]. In other words, hyperphosphorylated Tau can interfere with UPS function and further aggravate the development of AD. Given all the evidence above, it appears that UPS dysfunction could lead to the hyperphosphorylation of Tau and further promote the formation of NFTs. At the same time, hyperphosphorylated Tau may also affect the function of the UPS [105,111] and all these dysfunctions interact with each other, eventually leading to the occurrence of AD.

Synaptic plasticity is not only the ability of synapses to undergo morphological and functional changes in response to various stimuli, but it also has an important molecular basis for learning and memory. Moreover, the ubiquitin proteasome system plays a critical role in synaptic plasticity via the regulating of protein degradation, and defective proteolysis may cause the synaptic dysfunction observed in the early stage of AD [101,117]. Most of the available evidence has suggested that in neurons, ubiquitin-mediated-protein degradation is an important mechanism for modulating synaptic function and structure, and the process of neuronal connection very much depends on the balance of the UPS pathway [118]. Zhao et al. has found that the UPS can regulate the degradation of pre- and post-synaptic substrates thereby modulating synaptic plasticity, particularly in the brain of patients with AD. Meanwhile, the dysfunction of the UPS was shown to cause a failure in protein degradation and strongly inhibited synaptic plasticity, which was manifested by impairments of the long-term memory [119,120]. The UPS also plays a vital role in modulating the release of neurotransmitters and the reintegration of membrane receptors [89,120]. Particularly in neurons, as the key protein in the cAMP-dependent protein kinase A (PKA) signaling, CREB is directly involved in synaptic plasticity and cognitive function. Consequently, the activation of PKA and the phosphorylation of CREB are key mechanisms for memory formation [67,89]. In addition, the UPS can specifically bind to the regulatory subunit of PKA to promote its degradation and indirectly affect synaptic plasticity [121,122].

To sum up, the UPS serves as a major protein degradation pathway in eukaryotic cells, which regulates protein functions through multiple pathways and mechanisms. It is worth noting that the dysfunction of the UPS plays an important role in the occurrence and development of AD, and it may become a promising target for AD therapies. Therefore, deeply exploring the physiological functions of the UPS, while assessing its interrelationship with AD, will open a broader prospect for its application in drug research and development of AD treatments.

## 3. Current Treatment of AD

AD is one of the greatest healthcare challenges of the century. Especially as the global population continues to increase, so will the number of people affected by AD. It is predicted that the number of AD cases among the elderly will increase to 135 million by 2050 [123]. Currently, there are only four US Food and Drug Administration (FDA) approved drugs and one combination therapy available in the market for the symptomatic relief of AD [124], including the acetylocholinesterase inhibitors (AChEIs) based on cholinergic hypothesis (donepezil, galantamine, and rivastigmine) and noncompetitive N-methyl-d-aspartate (NMDA) receptor antagonist memantine [125]. Nevertheless, these medicines can only treat the symptoms, but they are inept at preventing the progression of the disease or reversing its influence [123]. Considering the current expectations of the increased number of AD cases each year and the huge financial cost amounted to health care, there is an urgent need to identify novel therapeutic targets and develop new therapeutic approaches in order to better manage AD [124].

In recent years, drugs targeting the pathobiological processes involved in AD have emerged [126]. These putative disease-modifying therapies aim to slow the progression of AD instead of only addressing its symptoms [126,127]. As previously noted, both Aβ and Tau are prime targets for disease-modifying treatments of AD. From this point of view, AD could be effectively prevented or treated by decreasing the production of Aβ and Tau, and by preventing, neutralizing or removing the aggregation and misfolding of these proteins [123,128]. Currently, therapies in trials targeting the amyloid cascade include agents aiming at decreasing amyloid-β production (β-secretase 1 inhibitors or α-secretase modulators) or increasing amyloid-β clearance (anti-amyloid-β antibodies or active immunotherapies) [127]. In addition to drugs targeting the amyloid cascade, drugs that target the Tau pathway (Tau aggregation inhibitors or anti-Tau antibodies) are being investigated [123,129,130] (Table 1); however, it is regrettable that despite many drug candidates having reached various clinical trial phases, most of the compounds did not succeed in Phase II/III trials due to adverse effects and a lack of therapeutic efficacy [124]. The high failure rate of AD treatment mainly stems from the complex pathologic causes of the disease, and the incomplete understanding of the relationships among the numerous pathways involved in the development of AD and subsequent neurodegeneration [131]. Therefore, it is imperative to understand the comprehensive pathogenesis of AD before focusing on novel drug development.

**Figure 5:**
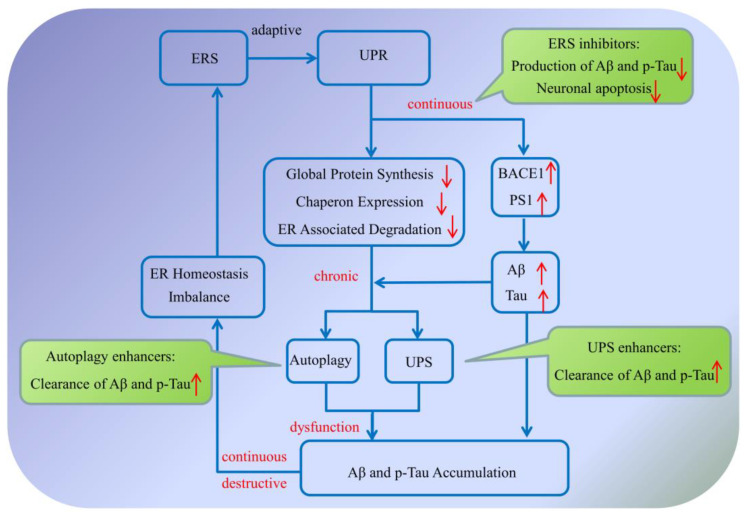
Related mechanisms and molecular targets between protein quality control system and Alzheimer’s disease. Aβ: amyloid-β protein; BACE1: β-amyloid precursor protein cleaving enzyme 1; ER: endoplasmic reticulum; PS1: presenilin 1; p-Tau: hyperphosphorylated Tau; UPR: unfolded protein response; UPS: ubiquitin-proteasome system. The upward red arrow indicates up-regulation of expression, while the downward red arrow indicates downregulation of expression. The blue V arrow indicates the activation of process.

There is no doubt that the protein quality control system plays an important role in the development of Alzheimer’s disease (Figure 5). On the one hand, when the protein quality control system is activated adaptively, it can effectively eliminate the abnormal accumulation of Aβ and p-Tau proteins in vivo, reduce the pathological changes of AD, and maintain the normal physiological activities and energy metabolism of cells [24]. On the other hand, when the body is under stress for a long time, the protein quality control system will change from adaptive activation to overactivation, thus breaking the homeostasis balance of the protein quality control system and even leading to cell apoptosis [55]. Meanwhile, it is important to shift the focus of AD drug development from treatment to prevention. Specifically, adaptive activation of the protein quality control system facilitates the clearance of abnormal proteins in the early stage of AD, while accumulation of misfolded substrates in the later stage of AD suggests an inadequate protein quality control or some failure to properly triage toxic protein substrates [132]. In turn, these chronic imbalances might impair the endoplasmic reticulum function, decrease autophagy efficiency, induce lysosomal dysfunction, and reduce proteasome activity, or even disrupt more global proteostasis. In this scenario, the proteostasis system eventually collapses, causing rampant aggregation of abnormal proteins and accelerating the development of AD [133].

Presently, a growing number of studies support the argument that the protein quality control system plays an important role in AD intervention. For example, memory impairment was recovered by removing brain-specific PERK expression in AD model mice [17]. ISRIB, as a widely expressed small molecule, has been reported to block the phosphorylation of eIF2α, which brought about the restoration of translation and improvement of long-term memory in rodents [134]. GSK2606414, an inhibitor of PERK, has the potential to block the Tau phosphorylation to improve neurodegenerative events [135]. Thus, selective inhibitors of PERK may serve as a candidate agent for the improvement of protein misfolding properties in neurodegeneration [136]. Meanwhile, dysfunction of the autophagic process impedes synaptic development and hampers axonal function, which might underlie the onset and progression of various neurodegenerative disorders [48], especially considering that neurons become highly susceptible to protein aggregation when autophagy responses are unable to eliminate the damaged protein or organelles effectively [133]. Therefore, enhanced autophagy may be another effective target approach for AD intervention. Additionally, the ubiquitination of proteins is precisely regulated by E3 ligases including Ubiquilin-1, CHIP, etc. Overexpression of Ubiquilin-1 alleviates cognitive deficits and reduces Aβ accumulation in mice [108] while CHIP has been found to contribute to the ubiquitination and degradation of several AD-related proteins, such as APP, Aβ, Tau, and BACE1 [115]. Therefore, improved CHIP levels can reduce the aggregation of Tau and the formation of NFTs, which may make it a candidate target for the treatment of AD [115].

## 4. Summary and Prospect

Taken together, we should fully realize that the protein quality control system has its special place in AD intervention. Future studies are warranted to further explore the interactions and communication between AD and the protein quality control system, especially regarding the cellular and molecular mechanisms including the external molecules and the internal signaling pathways that regulate nuclear mRNA transcription and protein translation. Furthermore, further studies can also combine animal studies and clinical research to overcome the shortcomings of animal AD models and the limitations in the materials of clinical research. In addition, to minimize the toxicity and side effects of current drugs, it would be another great challenge for more drugs with higher specificity and better selectivity to be developed based on the protein quality control system. For instance, naturally extracted plant compounds characterized by a multi-component and multi-target function may have certain positive effects in the intervention of AD. Meanwhile, selecting drugs that regulate the protein quality control network from the clinical use of compound preparations may also be a promising research direction for the treatment of AD. In addition, consideration should be given to the effects of non-pharmacological interventions that are known to affect the pathological process of AD, such as exercise, diet, social interactions, behavior modifiers, etc. Hopefully, future studies focusing on the protein quality control system will help to develop much needed novel therapeutics against AD.

## Figures and Tables

**Table 1:** Current pharmacological treatment of AD (clinicaltrials.gov accessed on: 29 November 2021).

Targets	Mechanism of Action	Drug/Clinical Trial	Status	Evaluation
Aβ	α-secretase modulators	Etazolate (EHT0202) [NCT00880412]	Phase II Completed	The agent was safe and well tolerated in patients with mild to moderate AD
	β-secretase inhibitors	LY2886721 [NCT01561430]	Phase I (Terminated)	Anomalous hepatic biochemical parameters of some participants were found
		Elenbecestat [NCT02956486]	Phase III (Terminated)	Unfavorable risk–benefit ratio including no evidence of potential efficacy, and the adverse event profile of being worse than placebo
		CNP520 [NCT02565511]	Phase II/III (Terminated)	Worsening of cognitive function in participants
		Verubecestat [NCT01953601]	Phase III (Terminated)	The decision to stop the study taken by the external Data Monitoring Committee
		Atabecestat [NCT02569398]	Phase II/III (Terminated)	Elevations in liver enzymes in subjects
	γ-secretase inhibitors	Semagacestat [NCT01035138]	Phase III (Terminated)	No clinical efficacy and skin cancer and some adverse reactions
		Tarenflurbil [NCT00380276]	Phase III (Terminated)	Low γ-secretase modulator potency
		Avagacestat [NCT00890890]	Phase II (Terminated)	Adverse effects: cerebral microbleeds, glycosuria and skin cancer
		NGP 555 [NCT02537938]	Phase I Completed	Not yet recruited in phase II study
	Reduction of Aβ-plaque burden	scyllo-inositol (ELND005) [NCT00934050]	Phase II (Terminated)	Did not provide evidence to support a clinical benefit of ELND005 while severe toxicity issues (infections) forced the cessation of the study
	Promotion of Aβ clearance (Active Aβ immunotherapy)	CAD106 [NCT00956410]	Phase II	CAD106 is an active Aβ immunotherapeutic agent
		ABvac40 [NCT03113812]	Phase I Completed	ABvac40 is evaluated in a phase 2 study, as the first active vaccine against the C-terminal end of Aβ 40
		GV1001 [NCT03184467]	Phase II Completed	GV1001 peptide (tertomotide) was previously studied as a vaccine against various cancers, whereas now it is evaluated in a phase 2 study for AD
		ACC-001 [NCT01284387]	Phase II Completed	ACC-001, an Aβ vaccine, was studied in phase 2a extension studies in subjects with mild to moderate AD
		UB-311 [NCT02551809]	Phase II Completed	A synthetic peptide used as an Aβ vaccine, has been advanced into an ongoing phase 2 study in patients with mild and moderate AD
		Lu AF20513 [NCT03668405]	Phase I (Terminated)	Lu AF20513 epitope vaccine is estimated in a phase 1 study in mild AD
Tau	Microtubule stabilizers	TPI-287 [NCT01966666]	Phase II	The agent was not well tolerated by the participants
		IONIS MAPTRx [NCT02623699]	Phase I Completed	The phase 2 clinical study is still in the recruiting process of patients with mild AD
	Targeting posttranslational modifications of Tau	Nilotinib [NCT02947893]	Phase II	It is now studied in a phase 2 trial in individuals with mild to moderate AD
	Inhibitors of Tau aggregation	Methylene blue [NCT00515333]	Phase III (Terminated)	Failed finally to show efficacy
	Promotion of Tau clearance (immunotherapy)	AADvac1 [NCT02579252]	Phase II	AADvac1 is currently studied in a phase 2 clinical study in mild to moderate AD
		ABBV-8E12 [NCT02880956]	Phase II	ABBV-8E12 is a humanized anti-Tau MAb assessed in a phase 2 clinical study in patients with early AD
		BIIB092 [NCT03352557]	Phase II (Terminated)	A phase 2 clinical trial assesses the safety and efficacy of the agent in participants with AD MCI and mild AD
		RO7105705 [NCT03289143]	Phase II	RO7105705 (MTAU9937 A) is an anti-Tau MAb which is assessed in a phase 2 study in individuals with prodromal and mild AD

Note: The date of last visit was 29 November 2021.

## Data Availability

Not applicable.

## Abbreviations

AD	Alzheimer’s disease
ALS	autophagy–lysosome system
ApoE	apolipoprotein E
APP	amyloid precursor protein
ASK1	apoptosis signal-regulating kinase 1
ATF4	activating transcription factor 4
ATF6	activating transcription factor 6
AVs	autophagic vacuoles
Aβ	amyloid-β protein
BACE1	β-amyloid precursor protein cleaving enzyme 1
β-CTF	β-carboxyl-terminal fragment
BDNF	brain derived neurotrophic factor
CHIP	carboxyl terminus of the Hsp70 interacting protein
CHOP	CCAAT/enhancer-binding protein homologous protein
CMA	chaperone-mediated autophagy
CREB	cAMP response element binding protein
DAPK1	death associated protein kinase 1
E1	ubiquitin-activated enzyme
E2	ubiquitin-conjugating-enzyme
E3	ubiquitin–protein ligase
ER	endoplasmic reticulum
ERAD	ER-associated protein degradation
ERS	endoplasmic reticulum system
GRP78	78 kDa glucose-regulated protein
HRD1	HMG-CoA reductase degradation protein 1
Hsp 70	heat shock protein 70
IRE1	inositol-requiring protein 1
JNK	c-Jun NH2-terminal kinase
LC3	microtubule-associated protein1 light chain 3
LSDs	lysosome storage disorders
NTFs	neurofibrillary tangles
PERK	protein kinase RNA-like ER kinase
PKA	protein kinase A
PS1	presenilin 1
p-Tau	hyperphosphorylated Tau
SPs	senile plaques
TRAF2	tumor necrosis factor receptor-associated factor 2
UCHL1	ubiquitin C-terminal hydrolase 1
ULK1	UNC-51 like kinase 1
UPR	unfolded protein response
UPS	ubiquitin-proteasome system
XBP1	X-box binding protein 1
